# A qualitative study of conflict experiences of Chinese physicians and nurses experiencing death of relatives: effects of dual roles

**DOI:** 10.3389/fpsyt.2025.1615027

**Published:** 2025-07-22

**Authors:** SiYing Xin, GuanMian Liang, QunFang Miao, Wei Lyu, SiYao Fan, HanYi Ning, JingXuan Zhang

**Affiliations:** ^1^ School of Nursing, Hangzhou Normal University, Hangzhou, Zhejiang, China; ^2^ Nursing Department, Zhejiang Cancer Hospital, Hangzhou Institute of Medicine (HIM), Chinese Academy of Sciences, Hangzhou, Zhejiang, China; ^3^ School of Clinical Medicine, Hangzhou Normal University, Hangzhou, Zhejiang, China; ^4^ Nursing Department, Dalian Women and Children’s Medical Center (Group), Dalian, Liaoning, China; ^5^ Nursing Department, The First Affiliated Hospital of Dalian Medical University, Dalian, Liaoning, China

**Keywords:** physicians and nurses, death, role conflict, qualitative research, Chinese

## Abstract

**Introduction:**

This study explored experiences of conflict among Chinese Physicians and Nurses following the loss of a loved one. This study aimed to examine the mechanisms behind these conflicting experiences as perceived by Chinese Physicians and Nurses.

**Methods:**

This qualitative exploratory study was conducted in three Chinese hospitals. Semi-structured in-depth interviews were conducted with 16 Physicians and Nurses members using a descriptive phenomenological analysis. Colaizzi’s seven-step analysis was used to analyze the interview data.

**Results:**

The following three themes and six sub-themes were identified: terminal stage (the responsibility exacerbates the burden of medical decision-making and professional expertise exacerbating internal conflict); acute loss stage (the experience of bereavement disrupts the professional values of medical personnel and emotional projection and avoidance of similar operations); bereavement recovery (reflection on the meaning of the profession and reconceptualization of death).

**Conclusion:**

These dual roles place significant psychological pressure on the Chinese Physicians and Nurses and lead to emotional fluctuations and complex internal conflicts when facing the death of their loved ones. Hospital administrators should recognize these contradictions, understand their complex emotions, and provide appropriate social support to address the needs of the Physicians and Nurses.

## Introduction

The death of a loved one is a significant source of stress for individuals (1), and studies have shown that bereaved individuals commonly experience significant emotional challenges, including loneliness, sadness, depressive symptoms, and diminished well-being (2). Western research on the emotional experiences of the bereaved has been conducted earlier, and more established theories have been developed to explain the emotional responses of the bereaved, such as Elizabeth Kubler-Ross’s Five Stages of Grief Model (3). These theories comprehensively summarize the emotional experience of the bereaved; however, some studies suggest that, due to cultural differences, Western theories may not fully apply to the traditional Chinese cultural context (4). Traditional Chinese culture is generally characterized by an optimistic, life-oriented perspective, emphasizing the value of living, and often avoids contemplating or discussing death. Traditional death taboos influence many Chinese individuals, and they are reluctant to engage with the topic of death, often advocating the concept of “better dead than alive.” (5) In Chinese culture, openly expressing grief is often seen as a sign of weakness, and individuals tend to suppress their grief, hindering emotional expression. Additionally, individuals who have lost a spouse or child in Chinese society may face stigma and even blame for the death, leading to social isolation and further stigmatization (6), this has contributed to a reluctance among Chinese individuals to discuss death openly. However, research has shown that the prolonged suppression of grief can result in serious mental health issues. Prolonged grief disorder is characterized by persistent and debilitating grief that lasts for one year or longer following a loss. Previous studies indicate that the prolonged repression of grief can lead to significant mental health issues (7). Although Physicians and Nurses regularly encounter death in their practice, it is assumed that they are adequately prepared to cope with such experiences. Nonetheless, studies have indicated that despite Physicians and Nurses’ generally accepting attitudes toward death, the overall situation remains less than ideal (8). Due to the influence of traditional death taboos and the slow development of death education in China, Physicians and Nurses continue to experience negative emotions, including fear, anxiety, and depression, when confronted with death (9).

In China, Shared Decision Making (SDM) is a commonly adopted healthcare model. In this model, Physicians and Nurses are required to assume the role of information providers, sharing detailed, disease-related information with patients and their families. They discuss available options, reach a consensus, and make informed medical decisions (10). Physicians and Nurses are expected to set aside personal opinions and adhere to the family’s preferences, even if those choices conflict with what the healthcare professional considers optimal. However, when confronted with the death of a loved one, Physicians and Nurses are no longer passive observers. Instead, they are required to make a critical decision, one that concerns the life of the loved one, creating a complex decision-making environment that induces significant conflict and internal contradiction for Physicians and Nurses. At the same time, society places high expectations on Physicians and Nurses, demanding that they maintain a calm, compassionate demeanor while providing meticulous care for each patient. However, when the patient is a loved one, the demands of medical ethics may no longer be the sole criterion guiding their decision-making. It remains uncertain whether such a conflict will lead to an intensified experience of internal struggle.

The concept of “roles” was first introduced by social psychologist George Herbert Mead (11) to examine patterns of individual behavior in social interactions (12). Roles represent the behavioral expectations that society holds for individuals with specific identities. In other words, there are established behavioral norms that dictate what individuals should and should not do in different roles (13), which reflect the ideal state of individuals’ behaviors and roles during the process of role construction. In social interactions, role conflict may arise between individuals due to divergent role expectations and behavioral patterns. Role conflict refers to the contradictions and conflicts that arise between or within individuals due to inconsistencies in role expectations, deviations in role perceptions, or inappropriate role behaviors during role enactment (14). Role conflict not only impacts the psychological well-being and interpersonal relationships of individuals but also affects the stability and development of society.

Current research on bereavement experiences predominantly focuses on the general population, with relatively few studies examining specific subgroups. This study aims to examine the mechanisms behind these conflicting experiences as perceived by Chinese Physicians and Nurses, who often occupy dual roles as medical professionals and family members during the death of a loved one. It seeks to uncover the underlying causes of such ambivalence from the perspectives of Physicians and Nurses themselves, thereby supporting hospital administrators in addressing their mental health needs. Additionally, the findings aim to inform the development of bereavement support policies tailored for Physicians and Nurses and aligned with traditional Chinese cultural values.

## Methodology

### Design and sample

This study adopted a descriptive phenomenological approach grounded in the philosophical framework of Edmund Husserl (15). Husserl emphasized that the core of phenomenology is to explore lived experiences as perceived by individuals, through a process known as “bracketing” (Epoché), which sets aside preconceived notions to faithfully describe the phenomena in consciousness, free from interpretation or theoretical imposition. As Husserl stated, phenomenology aims to “go back to the things themselves,” capturing and describing the essential phenomena of human experience. This theoretical foundation aligns with the aim of the present study, which is to capture the authentic emotional experiences of Physicians and Nurses coping with bereavement-related conflicts. Colaizzi’s seven-step analysis (16) was used to analyze, summarize, and refine the data. Physicians and Nurses members from three hospitals in China were selected using purposive sampling between January and February 2025.

Previous studies have indicated that bereavement within 12 months is considered an acute bereavement period (17). To avoid a more serious psychological impact on respondents during the interviews, this study included only participants who had experienced bereavement more than one year prior and asked them to review their past experiences. The inclusion criteria were as follows: (1) Relevant professional qualification certificate; (2) had experienced the death of a relative; (3) bereavement time ≥ 1 year; (4) volunteered to participate in this study. The exclusion criteria were as follows: (1) Physicians and Nurses unable to participate in the interview due to work, further study, or vacation; (2) Physicians and Nurses who could not clearly describe their emotional experiences.

The sample size was established based on the notion of data saturation, which is the stage at which no new themes, sub-themes, or significant deviations arise from the data. Saturation was assessed in accordance with accepted qualitative research criteria (18, 19), considering not only the frequency of themes but also the depth, diversity, and adequacy of the data. The data reached saturation after interviewing the 14^th^ participant, and two further interviews were conducted to confirm that no new details appeared. Sixteen Physicians and Nurses participated in these interviews.

### Sampling and recruitment

With the consent of the hospital administrators, three hospital workers who were also co-authors of this study posted recruitment posters on hospital bulletin boards without revealing their identities. Participants were recruited through self-registration. Sixteen medical professionals were interviewed for this study, including 13 females and 3 males, aged between 23 and 51 years (mean age 41.69 ± 7.34 years). Basic information about the respondents is provided in **Table 1**.

**Table 1:** Basic information about the interviewees.

Serial number	Sexes	Age	Profession	Deceased Loved Ones	Time of death
N1	Female	46	Nurse	Mother	2 years
N2	Female	23	Nurse	Father	1 year
N3	Female	48	Nurse	Husband	3 years
N4	Female	36	Physician	Mother	1 year and 3 months
N5	Female	51	Nurse	Mother’s Father	6 years
N6	Male	30	Nurse	Mother	2 years and 2 months
N7	Female	45	Physician	Mother	4 years
N8	Female	46	Physician	Sister	1 year and 4 months
N9	Female	48	Nurse	Husband	1 year
N10	Female	45	Physician	Father	1 year and 1 month
N11	Female	36	Nurse	Grandmother	3 years and 4 months
N12	Male	42	Physician	Mother	2 years and 5 months
N13	Female	46	Nurse	Younger Brother	2 years and 1 month
N14	Female	42	Nurse	Older Brother	1 year and 8 months
N15	Male	44	Physician	Wife	3 years and 7 months
N16	Female	39	Physician	Mother	1 year and 6 months

### Research tool

**(I) General information questionnaire**

A self-designed questionnaire was used to collect data on five demographic variables: age, sex, occupation, relationship with the deceased, and bereavement duration.

**(II) Interview outline**

Based on a literature review, a preliminary interview outline was developed after a thorough discussion among the research team members. Two medical professionals were selected for the pre-interviews. After further adjustments and refinements based on the pre-interview results and expert feedback, the final interview outline was established as follows: (1) How would you describe your emotional reactions from the time you lost your loved one to the present interview? (2) How do you manage your emotions? (3) During this period, did you experience conflict between your emotional and professional roles? Please elaborate. How did you address this conflict? (4) Did you notice any personal change during this time? (5) What kind of support did you receive from your family, friends, colleagues, or supervisors? How did this support help you? (6) What recommendations would you provide to healthcare institutions or society to help Physicians and Nurses cope better with the grief of losing their loved ones?

### Data collection

Before the interviews, the purpose, content, and methodology of the study were explained to the respondents. After informed consent was obtained, interviews were conducted at a convenient time for each participant. During the interviews, the environment was kept quiet and free of disturbances, and each session lasted between 45 and 60 min. With the participants’ permission, the interviews were audio-recorded, and the researchers listened attentively while also observing and recording the details of nonverbal communication. All interviews were transcribed within 24 h, and notes taken during the sessions were used to supplement the transcripts. After the transcription, the interview text was returned to the participants for verification to ensure the authenticity and accuracy of the information.

### Data analysis

This study adopted the descriptive phenomenological research method, grounded in the principles of Edmund Husserl’s phenomenology, and analyzed the data using Colaizzi’s seven-step analysis method (16).

First, the researchers began by setting aside preconceived beliefs and judgments about the bereavement experience. Second, they thoroughly familiarized themselves with and understood all the information provided by the participants by repeatedly and carefully reading the collected data. The transcribed text was imported into NVivo software, which was used to analyze the data word by word to identify and extract important and meaningful statements related to the bereavement experience. Third, the researchers coded the meanings of recurring ideas in NVivo, bracketing existing assumptions regarding bereavement experiences. Fourth, Following the identification of key meanings, the researchers grouped recurring ideas into related clusters. These clusters formed the themes. At this stage, the 19 significant meanings were categorized into six subthemes and three overarching themes. This step was driven by the data itself, ensuring that the themes were not pre-conceived but instead emerged naturally from the participants’ descriptions. Specific coding examples can be found in **Table 2**. Fifth, the researchers then provided detailed descriptions of each theme, exploring the underlying intentions behind each experience. This interpretation went beyond surface-level descriptions, focusing on the conflicts and emotional complexities experienced by physicians and nurses after the death of a loved one. The essence of each theme was elucidated by linking it to the broader emotional and psychological context, utilizing the participants’ verbatim statements to maintain the integrity of their lived experience. Sixth, the basic structure of the emotional experience of the Physicians and Nurses was described through an explanation of the themes, theme clusters, and verbatim quotes of the participants. Finally, the study results were presented to participants for verification to ensure that the findings accurately reflected their experiences. To prevent participants from reinterpreting or adding new layers to their experiences, we explicitly clarified that the purpose of member checking is to verify the accuracy of the data and to ensure that the researcher’s understanding of the participants’ narratives is correct, rather than revisiting or adding new interpretations. Participants are only asked to identify any factual errors or misunderstandings, and are not required to supplement or modify their original experiences. Furthermore, participant feedback is strictly limited to correcting potential misunderstandings or factual inaccuracies, without allowing for the reconstruction or addition of new elements to their experiences. In this study, the participants did not suggest any modifications after reviewing them.

**Table 2:** Examples of codes.

Primary node	Secondary node	Tertiary node	Example of reference point coding
terminal stage	the responsibility exacerbates the burden of medical decision-making	Stress from medical decision-making capacity conferred by relatives	I’m a doctor. My family depends on me.
		Work-related burdens exacerbate their stress	I go to work every day thinking about the family, which is actually quite dangerous now that I think about it.
	professional expertise exacerbating internal conflict	Choices made cannot be reconciled with learned ethical knowledge	If it were a patient, I’d tell them to give up, but this is my loved one, and I can’t stand by and watch him die.
		Lack of access to help exacerbates their suffering	I’ve asked every one of them, but they’re all hopeless.
		The feeling of powerlessness	God played a joke on me, I took a step and he blocked me.
acute loss stage	the experience of bereavement disrupts the professional values of medical personnel	Questioning medicine	Does studying medicine really help? I can cure all my patients, but I can’t cure my nearest and dearest.
		Loss of empathy	I don’t feel sorry for them, only for me.
		You can persuade others, but you can’t persuade yourself.	Those few words of comfort really pale in comparison, and I can’t even convince myself of that.
		Guilt persists	I regret every day if I made the wrong choice at the time.
	emotional projection and avoidance of similar operations	Fear, resistance to going to work	At first, I couldn’t even see the ward.
		Inability to perform similar medical operations	Those routine maneuvers I can’t do, I choose to avoid them, and my whole back is tingling.
		Regrets	I’ve been working for so long and have sent a lot of patients away, but the first one to pass away in front of me was my mom.
bereavement recovery	reflection on the meaning of the profession	The transformation of career values.	This incident has also had some impact on my career decisions in the future, maybe what I used to force, I don’t force very much now!
		Hopefully there will be new treatments available	I’ve since tried to learn about this area, so hopefully I can discover new treatments, I guess.
		Active volunteering	Commitment to some hospice-like volunteer activities
		help others	It’s the feeling of being indifferent to life and death.
		More empathy for patients	And I’m not just from the doctor’s point of view anymore, but also from the point of view of someone who’s been there.
	reconceptualization of death	Gained knowledge beyond books	That’s something you don’t learn in school.
		Fearless of death	accept life and death openly

### Data administration

All recordings were stored in secure electronic folders, accessible only to the research team, and were deleted after transcription.

### Ethical considerations

This study was approved by the Ethics Committee of Hangzhou Normal University (Approval No. 2024112) in December 2024. Before each interview, participants were provided with a detailed explanation of the research objectives and methods as well as the potential risks and benefits. Participation in this study was voluntary. None of the respondents chose to withdraw from this study. Each participant signed an informed consent form before the interview.

### Rigor and reflection

The interviewer was an experienced psychologist skilled in interviewing techniques, and all members of the research team were trained in qualitative research and interview methods. If any suspicious or inaccurate information arose during the interview, the interviewer sought verification from participants. All interview data were then shared with participants to confirm the accuracy and authenticity of their responses. Strict measures were taken to ensure the credibility, completeness, and accuracy of the data. The researchers carefully scrutinized the research process during data coding to objectively present the true intentions of the interviewees. Two researchers independently performed this process. The final coding and analyses were conducted through collaborative discussions among the research teams.

Additionally, neither the interviewers nor the data analysts in this study had a direct employment relationship with the sampled hospitals. During the project collaboration, three staff members from the sample hospitals participated in coordinating recruitment. To minimize the influence of the power dynamics arising from the investigators’ hospital positions on data collection, these three authors were strictly excluded from the core research processes, such as conducting interviews, data transcription, coding, and analysis. This structural role segregation was designed to ensure the authenticity and credibility of the interview data.

## Results

### Terminal stage

***The responsibility exacerbates the burden of medical decision-making.*** Respondents in this study emphasized the significant burden of medical decision-making placed upon them by their families due to their professional roles. This pressure originates not only from the decisions themselves but also from the expectations of family members and the respondents’ emotional struggles. When facing medical decisions during the terminal phase of a loved one’s life, Physicians and Nurses experience a strong internal conflict. Moreover, the overlap between professional obligations and family responsibilities often places them in an impossible situation where they are unable to fulfill both roles effectively. While work responsibilities require them to remain present in the hospital, their emotional focus is often drawn to family matters, leading to mental strain.

N3: “I studied medicine, so my family expects me to make the decisions. I knew the outcome, but when it’s my own loved one with so many eyes on me, it’s truly overwhelming. I didn’t even have anyone I could talk to. How could I explain it? I can lie to anyone, but I can’t lie to myself. It felt like every choice was the wrong one.”

N5: “During that period, I was thinking about everything happening at home every day, whether I was going to work or coming back. Looking back now, it was actually quite dangerous, but I couldn’t help it—it’s human nature. If I had been given more time off, it would have been better.”

***Professional Expertise Exacerbating Internal Conflict.*** Several interviewees indicated that the interplay between medical expertise and personal emotions intensified their internal conflicts and psychological burdens during end-of-life decision-making. Although their professional knowledge made them aware that the treatment’s efficacy was limited, their emotional attachment as family members made it difficult to let go. Rather than offering relief, their medical expertise heightened their awareness of the unresolved circumstances, contributing to emotional distress. Furthermore, they experienced a heightened sense of helplessness when they sought assistance but were unable to find effective solutions.

N6:”I knew there wasn’t much point in therapy at that moment, but I was going to do it.”

N7: “I consulted every classmate I knew, but none could offer help. It felt like God was playing a joke on me—every step I took, I encountered a new obstacle.”

N2 shared: “I thought I could save him with my expertise, but each solution led to a new problem. It felt like an endless maze.”

N1: “I could interpret the clinical indicators on the checklists. For patients, I would recommend withdrawal, but for my loved one, I couldn’t bear to watch him die. I had to try to save him. Applying what I had learned professionally to my own family felt utterly futile.”

### Acute loss stage

***The experience of bereavement disrupts the professional values of medical personnel.*** Many interviewees described experiencing intense psychological shock upon returning to their professional environment following the death of a loved one. Emotional numbness sharply conflicted with their professional responsibilities, affecting not only their clinical performance but also their capacity for patient empathy. The loss disrupted their emotional connection with patients and created a profound internal contradiction between personal grief and professional identity. In this collision between inner trauma and occupational role, Physicians and Nurses often become emotionally overwhelmed, eclipsing the needs of patients. During this time, Physicians and Nurses frequently contemplate whether their errors played a role in the death of their loved ones, a contemplation that has resulted in enduring sentiments of remorse and guilt.

N8: “During that initial period, I questioned whether studying medicine was even meaningful. I could cure all my patients, but I couldn't save the person closest to me.”

N12: “In the months after she passed, I had no empathy for my patients. I felt no pity for them—only for myself.”

N5: “Even though I knew all the right things to say, those words felt hollow when directed at myself. I couldned even convince myself. Years of faith felt meaningless. I questioned myself daily—was it all worth it? Did my choice of medical contribute to his death?”

***Emotional projection and avoidance of similar operations.*** During the acute grieving phase following the death of a loved one, some Physicians and Nurses exhibited pronounced emotional projection and avoidance of routine clinical procedures upon returning to work. The familiar medical environment and standard operations served as potent triggers for traumatic recollections. The overlap between their patients and the memory of their loved one's illness caused them to the trauma—not as medical professionals, but as emotionally reimmersed individuals. This disrupted their professional detachment and compromised their clinical judgment. Moreover, the emotional impact of the loss deeply interfered with their ability to integrate bodily perception with medical reasoning.

N9: “At first, I couldn’t even look at the ward. Seeing patients lying in bed made me feel like I was right back in that moment.”

N11: “After she passed, I returned to work and couldn't perform any routine tasks. I avoided them all, and my whole body would go numb.”

N7: “I have seen many patients die, but the first person to pass in front of me was my mother. I regret every day whether I made the wrong choice.”

### Bereavement recovery

***Reflection on the meaning of the profession.*** Some of the interviewees reported profound changes in their conceptions of death over time, which not only reshaped their ability to empathize with patients but also prompted them to reflect and sublimate the meaning of their profession in a more profound way. The personal experience of life and death became an important way for them to understand the pain and plight of patients and allowed them to gain a richer emotional and professional cognition of a deeper level of exploration and pursuit. Physicians and Nurses have expressed that this experience enables them to understand the inner world of patients and their families more deeply. The death of a loved one allowed them to get closer to the patient’s pain and enhanced the emotional perception and sense of responsibility of the Physicians and Nurses. The intrinsic drive for regret led them to hope that advances in medicine would save more people facing similar plights. Respondents also expressed a deeper awareness of life and death and wanted to pass on the strength they had drawn from their trauma to more people in need. The experience of life and death is not only a personal emotional sink but also inspires the motivation to provide support and care to others on a social level.

N6: “Slowly after a long time behind me, when I saw that kind of clinical reappearance of patients who had gone through similar experiences as my family, I realized that I could empathize with them more, and I felt very able to put myself in their shoes, and that kind of experience you can’t learn from a book.”

N10: “I’ve since tried to learn about this area, hopefully I can discover new treatments I guess, I may not be able to save my loved ones, but it’s comforting to myself to help some of the people who are suffering from the same disease.”

N8: “I’m now committed to volunteering in something like hospice care, anyhow, doing a little bit of what I can.”

***Reconceptualization of Death.*** Some of the interviewees slowly experienced a different feeling about the meaning of death after some time following the death of a loved one. These feelings did not come overnight but rather emerged slowly in daily life and internalization. Entering into that experience as an “experiencer” allowed the interviewees to move from abstract medical knowledge to embodied emotional resonance, and they gained a new understanding of their attitudes toward death.

N13: “Later on I put my feelings on some faith, and this thing has some influence on my career decision-making further on, maybe what I used to force, I’m not very forceful now, and it’s just a feeling of looking down on life and death.”

N4: “It feels like I can now accept life and death openly, and then when I face some late-stage patients, I no longer stand only from the doctor’s point of view, but also from the point of view of a person who has been there before, how can I say it, to provide some help for them as much as I can.”

## Discussion

### Dual roles trigger role conflict

The findings of this study indicate that Physicians and Nurses experience significant role conflicts when dealing with the death of a loved one. Specifically, when a loved one’s illness is terminal, the dual roles Physicians and Nurses occupy necessitate them to assume multiple responsibilities simultaneously, which can lead to overwhelming burdens, similar to the findings of previous research (20), work-related pressures and the heavy burden of caregiving contribute to increased stress among Physicians and Nurses. Furthermore, Physicians and Nurses often face emotional and rational conflicts when confronted with the death of a loved one. Feelings of helplessness arise from their inability to save their loved ones and their difficulty in adhering to the ethical principles of palliative care that they have been trained to follow (21), these factors exacerbate the dilemmas and conflicts faced by Physicians and Nurses, leading them to choose to “save even when they know it is hopeless.” At the same time, societal expectations project an image of omnipotence onto doctors and nurses, creating subconscious pressure to maintain a calm and objective demeanor, which may conflict with the emotional turmoil they experience. The resulting disturbances cause oscillations between these dual roles, generating role conflict and stress, which in turn intensifies anxiety, suffering, and internal burdens among Physicians and Nurses (22). Moreover, in addition to societal pressures, Physicians and Nurses are often burdened with the responsibility of medical decision-making for their loved ones, further complicating their ability to make rational decisions. Emotional attachments and the unrequited love they feel for their loved ones hinder their capacity to act in accordance with rational understanding. Previous studies have suggested that when individuals act in line with ethical norms, they tend to develop a sense of identification with their self-worth; conversely, deviating from these ethical norms can trigger profound feelings of shame (23). This implies that when Physicians and Nurses make decisions that contradict common sense, they not only exacerbate their moral dilemma but also experience heightened conflict. Moral distress (MD) refers to the psychological imbalance and emotional discomfort that arises when an individual’s actions conflict with their moral obligations and cannot be executed due to internal or external constraints (24). Previous research has also confirmed that moral dilemmas can have detrimental effects on Physicians and Nurses, such as mental health damage, burnout, and decreased job satisfaction (25).

### Dual roles deepen negative feelings of bereavement

The findings of this study suggest that Physicians and Nurses experiencing the death of a loved one often develop emotions such as grief and longing, similar to the bereavement experience outlined in previous research (26). Death is an unavoidable event in the human experience, but individuals’ attitudes toward death shape their understanding of life’s meaning (27). It is commonly assumed that Physicians and Nurses possess a deeper understanding of death and exhibit a higher level of acceptance of it compared to the general population, which may assist them in coping with the loss of their loved ones. However, the results of this study indicate that many Physicians and Nurses experience feelings of guilt, such as fear of decision-making in the face of a loved one’s death and questioning the value of their profession after the loss. These reactions may be attributed to the role of bereavement guilt. Previous studies also showed that among the different types of bereavement experiences, bereavement guilt is stronger among Physicians and Nurses. Bereavement guilt means the person feels bad about themselves because they think their relationship with the deceased did not meet their standards or expectations (28). Despite the role of guilt in maintaining the bereaved individual’s connection with their loved one from an evolutionary perspective (29), individuals with elevated levels of bereavement guilt caused by the irreparable disruption of the relationship may continue to self-blame for the death of the deceased, potentially developing negative beliefs that intensify their grief response (30). Physicians and Nurses often feel more guilt and self-blame when their loved ones die than other people. This may be because of their job, as Physicians and Nurses see saving lives and helping others as their purpose. Additionally, the lifelong professional values of Physicians and Nurses make them more likely to overestimate their responsibility for the death of their loved ones, leading to deeper self-reflection and increased feelings of guilt (31). Bereavement guilt compels the bereaved individual to constantly reexamine their decisions, exacerbating their guilt and creating a vicious cycle over time, which impedes emotional recovery.

### Emotional cumulative occupational effects

The results of this study indicate that Physicians and Nurses may feel as though they are re-entering the scene of their loved one’s death upon returning to the work environment. This phenomenon may stem from the fact that the healthcare worker’s grief has not been adequately processed. Flashbacks, one of the symptoms of post-traumatic stress disorder (PTSD), refer to sudden, vivid recollections of a traumatic event, which can make the individual feel as if they are reliving the traumatic experience. Intense emotional reactions, such as fear, hopelessness, or anxiety, frequently accompany flashbacks. These fragmented memories can suddenly appear during daily activities, which harms a person’s mental health and quality of life (31, 32).Physicians and Nurses, unlike other workers, often have to return to clinical settings after a loss. Bereavement leave in China is short, and being exposed again to clinical environments that remind them of a loved one’s death in a short time can be a form of repeated trauma. This, in turn, significantly increases the likelihood of persistent traumatic stress reactions among Physicians and Nurses. This condition, known as Complex Post-Traumatic Stress Disorder (C-PTSD), arises from prolonged exposure to traumatic events or intense stress. Symptoms of C-PTSD include anxiety, depression, self-denial, fear, helplessness, anger, and cognitive dissonance regarding self and others, Additionally, relationship problems may arise (33). The cumulative effect of these factors can result in the emergence of negative emotions in Physicians and Nurses, which, unfortunately, are often difficult to detect and are frequently overlooked by both Physicians and Nurses and their managers. This neglect can harm the mental health of Physicians and Nurses and lead to empathy fatigue and burnout. These conditions can affect their professional values. Studies show that Physicians and Nurses’ values are linked to the quality of care, patient satisfaction, and even patient safety (34). Hospital administrators need to focus on the mental health of Physicians and Nurses after they lose a loved one and improve support for them.

### Reconstruction and elevation of the meaning of occupation

The results of this study show that Physicians and Nurses, after losing a loved one, better understand life and death. This change may come from the deep personal pain of losing someone, which makes them think more about the meaning and value of life. This thinking helps improve their spirituality. Spirituality is a natural part of everyone’s mind. It represents a spiritual potential (a force transcending rationality and emotion) that is embedded within every individual, reflecting a deep yearning for the meaning and value of life. It also represents the expression of the human spiritual realm as it continuously evolves and develops (35).

The findings of this study suggest that some Physicians and Nurses choose to adjust their life plans and strengthen their professional knowledge after the death of a loved one. This may be attributed to the enhancement of their spirituality, which allows them to view life from a broader perspective (36). By deepening their knowledge in order to save more lives, they align with previous research, which shows that spirituality can intrinsically motivate individuals to fulfill their spiritual needs based on a sense of calling and role membership. The challenging nature of creative activities further inspires intrinsic motivation in individuals (37). Through continuous learning, Physicians and Nurses improve their knowledge and empathy. This matches transformative learning theory, which focuses on education that centers around the learner through critical thinking and discussion (38). This method encourages learners to question their beliefs, leading to a deeper understanding and a change in perspective that guides their actions (39). When Physicians and Nurses experience the loss of a loved one, they better understand life and death, which helps them empathize with patients’ emotions. This understanding also motivates them to learn more and fully engage in their work. The results of this study also suggest that Physicians and Nurses may choose to volunteer for hospice and other organizations after experiencing the death of a loved one. In doing so, they aim to reconstruct their sense of professional significance by helping others who share a common experience. This matches earlier research (40), which found that Physicians and Nurses who have faced personal and professional loss are better at patient- and family-centered communication and care. So, Physicians and Nurses should be encouraged to connect their personal and professional experiences related to death and dying, as this can help them do their jobs better.

### Construction of intervention strategies

The results of this study reveal that Physicians and Nurses undergo complex emotional shifts and multiple emotional shocks after experiencing the death of a loved one. However, in general, most Physicians and Nurses achieve personal growth, although this process requires a significant amount of time and support. Hospital administrators should focus on creating a complete support system for Physicians and Nurses. For example, meaning-centered psychotherapy can help Physicians and Nurses deal with different psychological problems (41). Support groups, where people with similar experiences come together, can be organized to share their stories and give emotional help. Some studies have demonstrated that shared experiences have a unifying effect. Despite differences in members’ backgrounds and family situations, the common experience of losing a loved one fosters a close-knit group that develops a shared culture and narrative through the exchange of personal stories (42). This group provides a safe space where members can accept behaviors that may seem “strange” to outsiders, thereby redefining the concept of “normalcy” after bereavement.

Additionally, extended bereavement leave and policy support, such as bereavement support groups in other countries (43), should be provided to ensure better bereavement support for Physicians and Nurses. Furthermore, life and death education courses for Physicians and Nurses should be strengthened. Life and death education, as part of medical humanities courses, encourages respect for life and helps Physicians and Nurses confront death directly. It aids them in developing a correct outlook on life and death (44), understanding the nature and significance of death, and cultivating a healthy attitude toward it.

### Strengths and limitations of the work

This study used in-depth interviews to explore Physicians and Nurses’ experiences of conflict before and after losing a family member. The goal was to understand how these conflicts are shaped from the workers’ perspective and help hospital administrators improve bereavement support. There are some limitations to note. First, the participants were Physicians and Nurses who kept working after the death of a loved one. These workers showed strong emotional resilience and were able to do their jobs well despite the traumatic event. Second, the study had more female participants, which means the impact of gender differences on the findings was not fully explored.

### Recommendations for follow-up studies

Future research could include the views of people who have left the healthcare field to study the long-term effects of bereavement on Physicians and Nurses using a long-term research method. This would give healthcare managers a better basis to improve bereavement support programs. Also, the sample size in this study was small, with few healthcare organizations and few male Physicians and Nurses. Future studies could adopt quantitative methods to include a larger male sample and systematically track the developmental trajectory of conflict and cognitive discrepancies among healthcare practitioners who experience family bereavement. Future research could investigate how the distinct professional experiences of physicians and nurses influence their experiences of bereavement. Understanding these differences may provide valuable insights into profession-specific support strategies and improve targeted interventions for Physicians and Nurses coping with personal loss.

## Conclusion

In summary, Physicians and Nurses undergo complex emotional changes when experiencing the death of a relative. Physicians and Nurses may have some ways to cope with death, but their jobs cause them to feel stronger emotions when they lose someone close. A good support system should be set up for Physicians and Nurses. This could include giving more time off for grieving and creating support groups where they can help each other. This study has several limitations. First, the participants were Physicians and Nurses who continued working after experiencing the death of a loved one. These workers managed to adapt to the hospital environment after the traumatic event and demonstrated positive emotional adjustment. The study mainly focused on female perspectives and did not consider how gender might affect the bereavement experience. Future research could look at both male and female Physicians and Nurses, as well as those who have left their jobs. It could also use a long-term approach to study the lasting effects of bereavement. This would give better information for creating support policies for hospital administrators.

## Data Availability

The raw data supporting the conclusions of this article will be made available by the authors, without undue reservation.
